# The year in cardiovascular medicine 2021: arrhythmias

**DOI:** 10.1093/eurheartj/ehac007

**Published:** 2022-02-07

**Authors:** Harry J.G.M. Crijns, Prashantan Sanders, Christine M. Albert, Pier D. Lambiase

**Affiliations:** Department of Cardiology and Cardiovascular Research Centre Maastricht (CARIM), Maastricht University Medical Centre, Maastricht, The Netherlands; Centre for Heart Rhythm Disorders, University of Adelaide and Royal Adelaide Hospital, Adelaide, Australia; Department of Cardiology, Smidt Heart Institute, Cedars Sinai Medical Center (CMA), Los Angeles, CA, USA; Department of Cardiology, University College London and Barts Heart Centre, London, UK

**Keywords:** Guidelines, Randomized trial, Public health, Societal impact, Atrial fibrillation, Ventricular arrhythmias, Brugada syndrome, Implantable defibrillator, Cardiogenetics

## Abstract

**Graphical Abstract:**
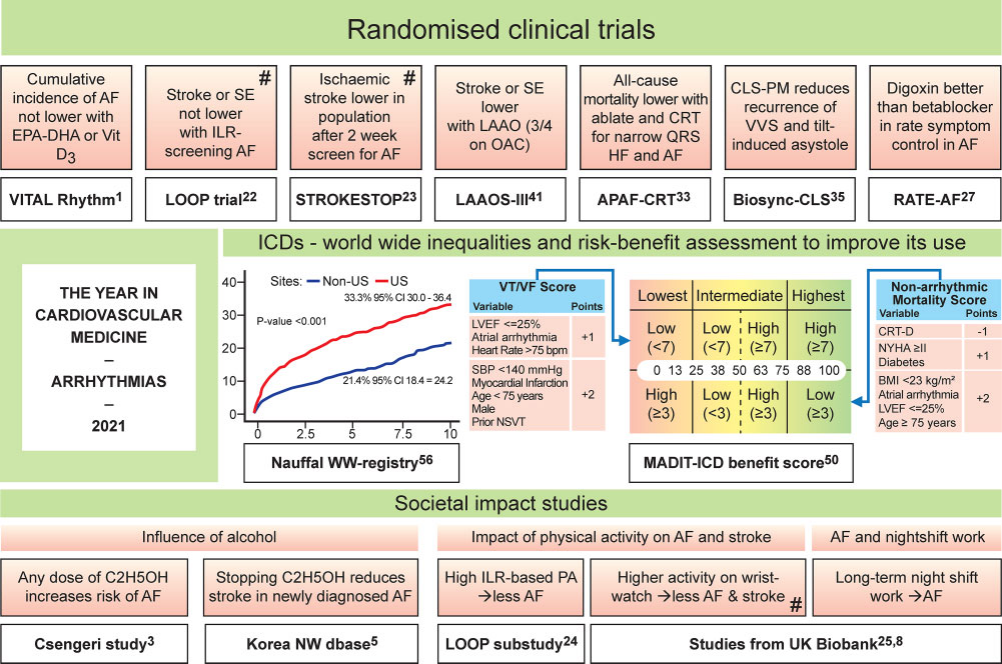
Randomized trials reported on food supplements to prevent atrial fibrillation (AF),^1^ screening for AF,^22^ and left atrial appendage occlusion^41^ to prevent stroke and novel pacing strategies to prevent death in heart failure patients^33^ or syncope recurrence.^35^ In RATE-AF, digoxin was superior to bisoprolol,^27^ illustrating an old drug can be effective if wisely applied with a patient-oriented endpoint. To improve the impact of primary prevention ICD, the MADIT-ICD benefit score balances the risk of sudden cardiac death and the competing risk of non-arrhythmic death^50^ (calculator at https://redcap.urmc.rochester.edu/redcap/surveys/index.php?s=3H888TJ8N7). The worldwide differences in ICD usage^56^ further support a unified approach focusing on ICD benefits. Contrary to current guidelines, the EAST-AFNET4 substudy suggests that (early) rhythm control benefits asymptomatic and symptomatic patients alike concerning cardiovascular endpoints.^57^ Alcohol does not protect from AF no matter dose or type of alcohol (Csengeri study),^3^ although the latter is at variance with another recent BIOBANK study.^7^ Stopping consuming alcohol after detection of AF may reduce stroke;^5^ it may also reduce the recurrence of AF after ablation.^6^ Less AF^24,25^ and stroke^25^ was also seen with higher levels of physical activity (PA) as measured by modern day monitoring technology (#) in LOOP trial^24^ and UK Biobank.^25^ Also from the UK Biobank: long-term night shift work may cause AF.^8^

Randomized trials reported on food supplements to prevent atrial fibrillation (AF),^1^ screening for AF,^22^ and left atrial appendage occlusion^41^ to prevent stroke and novel pacing strategies to prevent death in heart failure patients^33^ or syncope recurrence.^35^ In RATE-AF, digoxin was superior to bisoprolol,^27^ illustrating an old drug can be effective if wisely applied with a patient-oriented endpoint. To improve the impact of primary prevention ICD, the MADIT-ICD benefit score balances the risk of sudden cardiac death and the competing risk of non-arrhythmic death^50^ (calculator at https://redcap.urmc.rochester.edu/redcap/surveys/index.php?s=3H888TJ8N7). The worldwide differences in ICD usage^56^ further support a unified approach focusing on ICD benefits. Contrary to current guidelines, the EAST-AFNET4 substudy suggests that (early) rhythm control benefits asymptomatic and symptomatic patients alike concerning cardiovascular endpoints.^57^ Alcohol does not protect from AF no matter dose or type of alcohol (Csengeri study),^3^ although the latter is at variance with another recent BIOBANK study.^7^ Stopping consuming alcohol after detection of AF may reduce stroke;^5^ it may also reduce the recurrence of AF after ablation.^6^ Less AF^24,25^ and stroke^25^ was also seen with higher levels of physical activity (PA) as measured by modern day monitoring technology (#) in LOOP trial^24^ and UK Biobank.^25^ Also from the UK Biobank: long-term night shift work may cause AF.^8^

The year 2021 yielded remarkable societal impact arrhythmia papers reporting on important public health issues, the latest ESC 2021 pacing guidelines, randomized trials on atrial fibrillation (AF) and cardiac pacing, and intriguing multidisciplinary aspects of AF, with progress in ventricular arrhythmias, in particular an outstanding series of Brugada syndrome (BrS) studies.

## Public health and societal issues matter in atrial fibrillation

A range of papers indicates that foods and food supplements, health behaviours, work and sleep environment, and life events may increase the incidence of AF in turn affecting health in the population and drawing attention to the need for reform. Dietary supplements were investigated in the VITAL Rhythm Study (*Graphical Abstract*).^1^ Incident AF was not significantly reduced over 5.3 years by either omega-3 fatty acids or vitamin D supplementation. Indeed a meta-analysis of randomized trials in patients with increased vascular risk showed that supplementation with marine omega-3 fatty acids increases the 1.2% yearly risk of incident AF by 25%, especially if >1 g/day is ingested to be discussed with your patient when optimizing AF management.^2^ The mechanisms remain an area of future exploration.

**Figure 1:**
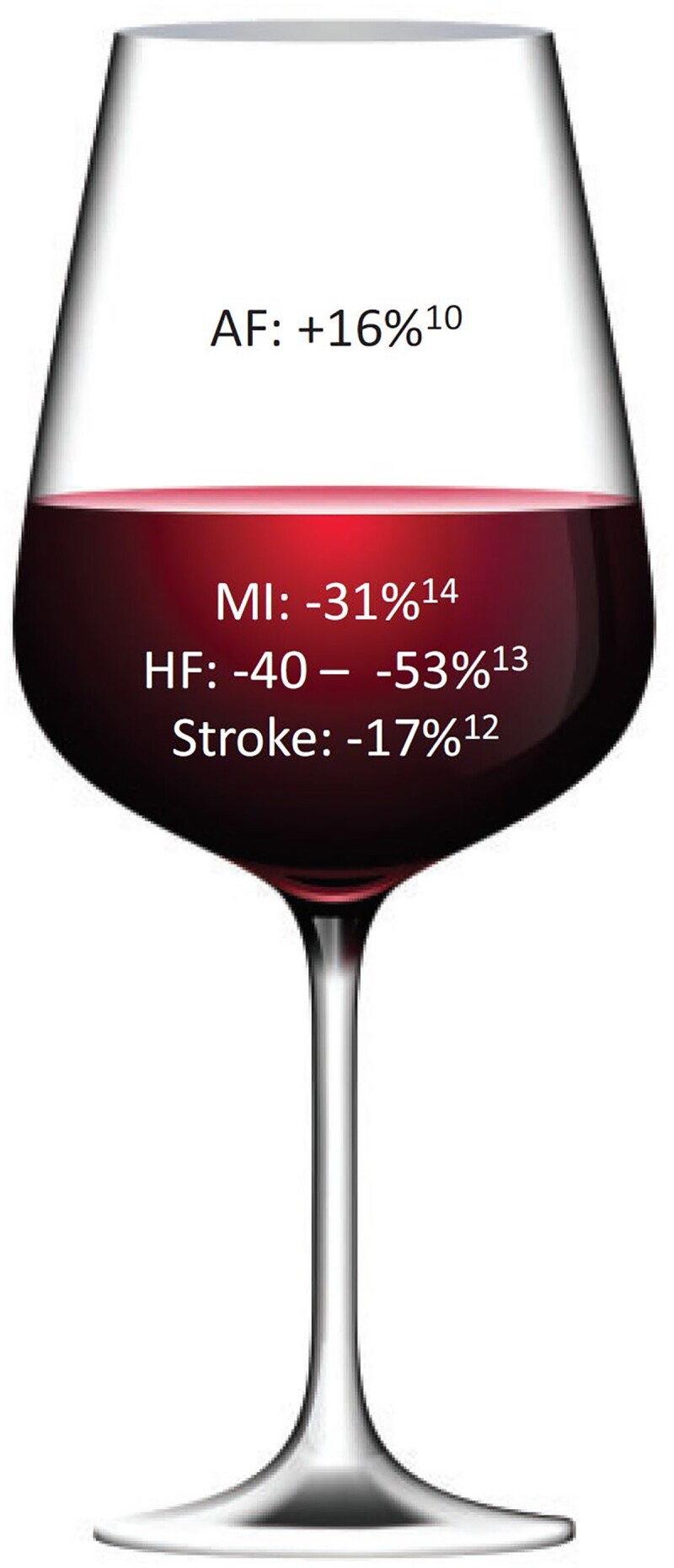
Alcohol consumption and risk of cardiovascular associations per one standard drink: finding the right balance. Reproduced with permission from Wong and Conen.^4^

Daily alcohol consumption of one standard drink is long said to be protective in myocardial infarction, heart failure, and stroke but did not protect from new AF despite how low the alcohol dose was in 107 845 individuals in five prospective community-based cohorts (*Figure 1*).^3,4^ Furthermore, abstinence from alcohol after a new diagnosis of AF was associated with a 14% reduction in stroke compared with continued drinking in a population-based study from Korea.^5^ So, alcohol and AF seem to have an atypical relationship^6,7^ vs. other cardiovascular disorders.

The incidence of AF is also impacted by social stressors with studies demonstrating increases in AF risk among night shift workers, regardless of their genetic background risk for AF.^8^ Parents losing a child also have an on average 15% increased AF risk, especially in the first week after the loss, with atrial arrhythmogenic sympathetic arousal, substance use, inflammation, or the renin–angiotensin–aldosterone system as mediating factors.^9,10^ Sleep arousal burden is associated with long-term all-cause and cardiovascular mortality in community-dwelling elderly, but unknown if it increases the incidence of AF.^11^

## Resuscitation

From the Swedish Register for Cardiopulmonary resuscitation, one paper showed that low socioeconomic status reduces chances of survival after in-hospital cardiac arrest due to lack of rhythm monitoring and delayed CPR,^12^ in line with out-of-hospital cardiac arrest.^13^ Socioeconomic prejudices leading to inappropriate health inequalities demand re-engineering societal conditions.^12,14^ A second paper from this registry reported decreased 30-day survival from 9.8 to 4.7% in out-of-hospital arrests in patients suffering from COVID-19 compared with non-COVID-19 arrests, and from 39.5 to 23.1% for in-hospital cardiac arrests, respectively.^15^ Although this may relate to the early COVID-19 recommendation from the authorities to avoid bystander ventilation, arrests were more often associated with non-shockable rhythms and pulmonary failure. At the same time, due to COVID-19 restrictions, an unexpected 32% reduction in ventricular arrhythmias needing device therapies was reported,^16^ while the converse of a 33% increase pacemaker/ICD detected atrial arrhythmia episodes was found in otherwise stable rhythm device patients.^17^

A novel strategy of alert system-supported lay defibrillation and basic life-support was superior to usual resuscitation in a randomized study from the Netherlands with improved out-of-hospital cardiac arrest survival from 26 to 39% and 50% more patients with neurologically favourable outcome.^18^ A spectacular new concept from Sweden concerned drone delivery of an AED to the out-of-hospital cardiac arrest scene integrated in the standard emergency medical services, showing that delivery was feasible with earlier arrival of AED by 2 min.^19^ Both papers illustrate that the chain-of-survival for cardiac arrest is boosted significantly by novel technologies and implicitly they provide an example for regions with less advanced resuscitation infrastructure.

## New pacemaker and resynchronization guidelines

**Figure 2:**
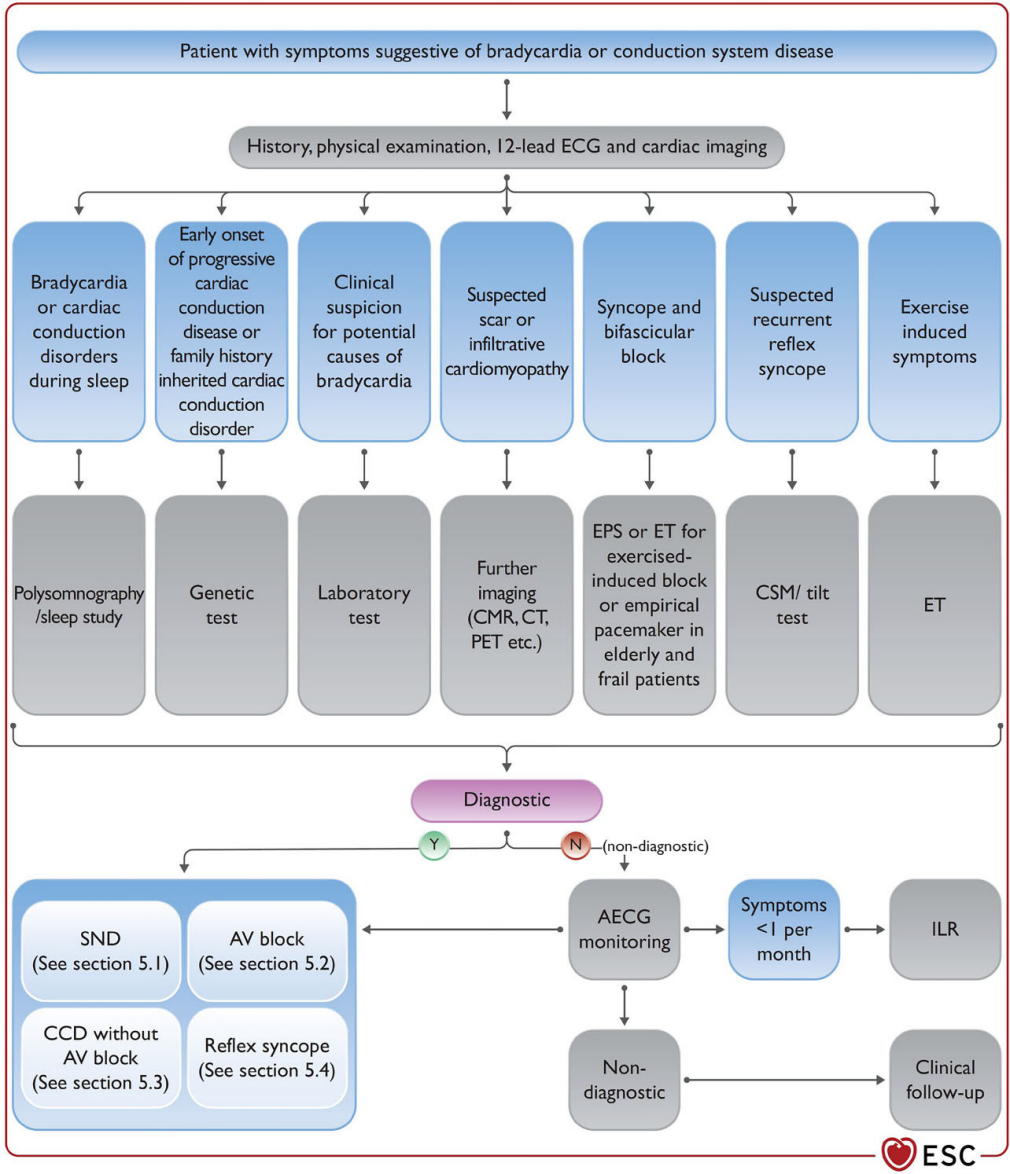
One of the didactic figures from the ESC 2021 Guidelines on cardiac pacing and resynchronization explaining the evaluation of bradycardia and conduction disease. Reproduced with permission from Glikson et al.^20^

The 2021 ESC Guidelines on cardiac pacing and resynchronization therapy, updated from 2013, address many new areas including pacing in TAVI, conduction system pacing, novel insights into cardiac resynchronization therapy (CRT) indications, and leadless pacing.^20^ Figures summarizing management in the increasingly complex areas of seemingly simple conditions like suspected bradycardia or conduction system disease are provided (*Figure 2*). A long list of gaps in evidence is outlined as an invitation to perform randomized trials and observational big data studies. Gaps that could be addressed include optimal pre-implant programming, prediction of pacing-induced cardiomyopathy, long-term effects of conduction system pacing, prediction of AVB after TAVI, and acute device implantation in patients with an active infection. The Guidelines also highlight evidence gaps in the effects of patient education, patient-centred care, and shared decision-making.^20^

## Randomized controlled trials in atrial fibrillation with or without heart failure

Screening for AF is currently recommended on an opportunistic basis for patients over the age of 65, using pulse palpation or ECG rhythm strip.^21^ The LOOP study investigated a more strategic screening approach, utilizing Medtronic LinQ^®^ loop recorders for continuous heart rhythm monitoring.^22^ Elderly patients were randomized to loop recorder implant for AF screening or standard care. Anticoagulation was started for patients in whom >6 min of AF was detected. Subclinical AF was more frequently diagnosed in the loop recorder arm, but anticoagulation of these patients did not result in a significantly reduced incidence of stroke or systemic arterial embolism over >5 years. Similarly, another randomized AF screening study published this year, STROKESTOP, which utilized a less intensive screening involving twice-daily 12-lead ECGs for 14 consecutive days in a larger population of 75–76-year olds, also did not find a significant benefit on ischaemic stroke compared with usual care.^23^ However, this study did report a significant modest 4% reduction conferred by AF screening on the primary composite outcome of ischaemic or haemorrhagic stroke, systemic embolism, major bleeding leading to hospitalization, or death compared with usual care.^23^ Therefore, while strategic screening programmes for AF may be of use in elderly patients, there do not appear to be marked benefits on ischaemic stroke in unselected patients. Also, the specific role of long-term continuous monitoring and the precise burden of AF required to instigate anticoagulation remains unclear. A separate LOOP analysis assessed physical activity measured through the loop recorder showing that a 1 h reduction in average daily physical activity was associated with an increased odds of AF onset the next day by ≈25%,^24^ a finding supported by a recent UK Biobank analysis using a wrist-based accellerometer.^25^ These data strengthen the evolving links between modifiable risk factors, physical activity, and AF providing a foundation for new tools to evaluate and intervene in lifestyle modification programmes.^26^

RATE-AF compared bisoprolol with low-dose digoxin as first-line therapy in permanent AF and high ventricular rates.^27^ Both drugs were found to be equally effective in reducing resting heart rate and there were no differences between the two arms in patient-reported quality of life at 6 months. Furthermore, digoxin was associated with fewer adverse events, suggesting that digoxin may have a place as an alternative to beta-blockade as first-line therapy despite previous observational studies suggesting increased mortality.^28–30^

The APAF-CRT trial compared medical rate control with a pace and ablate strategy^31,32^ using CRT as opposed to right ventricular pacing in patients with permanent AF, heart failure, and narrow QRS on ECG.^33^ AVN ablation and CRT were superior to medical rate control, resulting in a 74% reduction in all-cause mortality and a 60% reduction in heart failure hospitalization. These substantial reductions are compelling and highlight the utility of this strategy over medical rate control in a specific cohort of elderly patients with heart failure and permanent AF.

## Syncope

Investigation and management of patients with recurrent reflex syncope remain a significant clinical challenge. The utility of tilt-table testing in diagnosis has been questioned, but a state-of-the-art review^34^ highlighted its advantages, which include the ability to correlate symptoms, blood pressure, and heart rhythm, providing the ability to assess the temporal association between bradycardia and syncope optimize the selection of patients who may benefit from pacing. Brignole *et al*.^35^ studied patients over 40 years old with recurrent reflex syncope and tilt-induced syncope with an asystolic pause longer than 3 s who had dual-chamber pacemakers with closed-loop stimulation (CLS) function. Patients were then randomized to either active (‘pacing on’) or inactive (‘pacing off’). The ‘pacing on’ group had a 77% reduction in risk of recurrent syncope compared with the ‘pacing off’ group, highlighting both the clinical utility of tilt-induced asystole and the efficacy of pacing with CLS.

## Multidisciplinary atrial fibrillation

Chronic obstructive pulmonary disease shares common risk factors with AF and may cause AF-genic atrial structural remodelling and increased sympathetic nerve activity (the latter also boosted by beta-agonists), caused by hypoxaemia and hypercapnia, increased thoracic pressure swings, systemic inflammation, and accelerated ageing.^36^ Chronic obstructive pulmonary disease in AF patients associates with sleep apnoea, heart failure, coronary disease, and diabetes. Chronic obstructive pulmonary disease contributes to AF progression and recurrences after rhythm control therapies, increases the risk of all-cause and cardiovascular death, stroke, and major bleeding in AF patients, and therefore requires a multidisciplinary management approach.^36,37^

Verdonschot *et al*.^38^ clustered dilated cardiomyopathy (DCM) in four phenotypes, one of which is the arrhythmia DCM-phenocluster mainly consisting of AF and pointing to either a common mechanism leading to DCM and AF (with atrial failure as one of the presumed linking mechanisms) or AF causing reversible tachycardiomyopathy. In a Mendelian randomization study, AF was found to be a causal factor for renal impairment rather than the reverse.^39^ Presumed linking mechanisms are haemodynamic or thrombo-embolic, but whether elimination of AF would reduce the incidence of kidney failure is as yet uncertain.^40^ In the LAAOS-III trial, removal of the left atrial appendage during cardiac surgery reduced the risk of stroke in patients continuing oral anticoagulation^41^ and should be considered in all cardiac surgeries in high-risk AF patients.^42^ Notably, one-quarter of NOAC users appear to discontinue the drug leading to avoidable strokes,^43^ but LAAOS-III does not address stand-alone appendage closure in non-compliant patients.

Utilizing serially assessed hsTnT and NT-proBNP improves the ABC stroke risk score and the same holds for GDF-15 incorporated in the ABC bleeding risk score.^44^ Although this paper supports the notion that serial biomarkers may better reflect the risk of adverse events in AF,^21^ it may not immediately change practice: what if NT-proBNP and hsTnT increased in an already anticoagulated patient? Add platelet inhibitor and put in an appendage occluder? Or reduce anticoagulation and manage already well-managed bleeding risk factors in the case of an increase in GDF-15?^45^ By design, since all patients were CHA2DS2-VASc 2 or greater and treated with anticoagulation, the study could not answer whether the serial assessment of biomarkers might help to identify patients at apparently low risk by CHA2DS2-VASc who might benefit, and randomized clinical trials are dearly needed in this area. For the time being, for the low-risk AF patients, an easy to use decision tree for or against adding anticoagulation can be found in Sulzgruber *et al*.^46^

## Ventricular arrhythmias and sudden cardiac death

**Figure 3:**
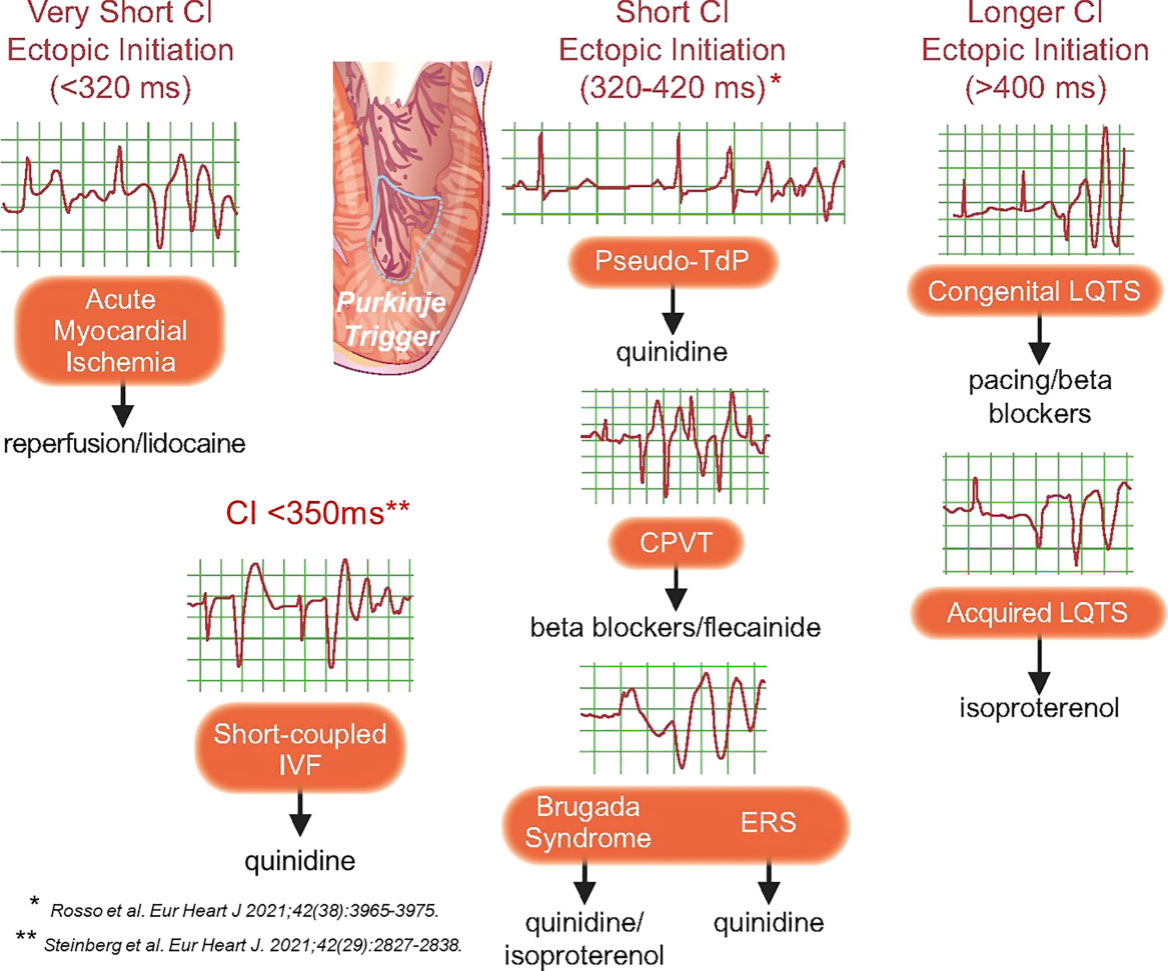
Coupling intervals at the initiation of various ventricular arrhythmias including short-coupled variants.

Studies in this area include elegant clinical observations from the humble surface ECG, epicardial mapping, to functional genetic studies. Two specific papers focus on the initiation of ventricular fibrillation (VF). Viskin and colleagues^47^ examined the behaviour of triggering ventricular ectopics in 87 patients with coronary artery disease who developed spontaneous polymorphic ventricular tachycardia (VT) responsive to quinidine therapy in the absence of ischaemia. In 32 patients, the QT interval was prolonged. However, when comparing the polymorphic VTs of these patients, which were termed ‘pseudo-Torsade de Pointes (TdP)’, with 53 patients with true TdP in the context of drug-induced LQTS, they noted that the coupling interval of the initiating ectopic beat was shorter than 400 ms in pseudo-TdP and (much) longer than 400 ms in true TdP. In addition, the QT interval in pseudo-TdP was shorter, the mode of onset was less often pause dependent, and the initial R–R intervals were shorter than in true TdP. Finally, patients with pseudo-TdP responded well to quinidine therapy, whereas quinidine is obviously detrimental in true TdP. Thus, in patients with pseudo-TdP, polymorphic VTs occur in the presence of a prolonged QT interval, but not due to a prolonged QT interval.^47^ These observations have important implications in managing polymorphic VT/VF in coronary artery disease patients to identify ‘quinidine responders’^48^ (*Figure 3*).

The CASPER investigators described a distinct novel phenotype of idiopathic VF initiated by a trigger PVC with a coupling interval of <350 ms, short-coupled ventricular fibrillation (SCVF).^49^ Among 364 unexplained cardiac arrest (UCA) survivors, 6.6% met diagnostic criteria for SCVF. Electrical storm occurred in 21% of these probands but not in other UCA probands (*P* < 0.001). Recurrent VF resulted in quinidine administration in 50% SCVF cases with excellent arrhythmia control indicating this should be a first-line treatment. The lesson learned from both these studies is to carefully evaluate VF initiation examining the coupling intervals of the initiating beat as this can have critical implications for polymorphic VT/VF drug management using quinidine (*Figure 3*).

Over the past year, there has been increased recognition of the importance of considering competing risks of mortality when considering who might benefit from ICD therapy. The MADIT-ICD score quantified the risk of cardiac arrest vs. other competing causes of death and reported on separate prognostic score models for VT and non-arrhythmic mortality.^50^ The two scores were combined to create three MADIT-ICD benefit groups. In the highest benefit group, the 3-year predicted risk of VT/VF was three-fold higher than the risk of non-arrhythmic mortality (20 vs. 7%, *P* < 0.001). This personalized benefit score predicted the likelihood of prophylactic ICD therapy weighed against the risk of non-arrhythmic mortality enabling a more informed discussion with patients.

In a risk stratification study focusing on BrS, the Shanghai Brugada diagnostic score was compared with the Sieira score (which combines a number of risk factors including Type 1 resting Brugada ECG, family history of sudden death, and inducibility of VT/VF at EP study): both scores differentiate equally between high- and low-risk patients but perform equally poorly for intermediate-risk cases.^51^ Interestingly, there were very few sudden deaths with an overall risk of 0.15% per annum, i.e. equivalent to the general population indicating that although risk assessment in BrS needs to be refined, very few sudden deaths occur using current individual clinician-based risk stratification strategies;^52^ however, recent studies suggest that genetic profiling may identify higher-risk subgroups.

To this end, Ishikawa *et al*. demonstrated that the loss of function (LOF) SCN5A mutation carriers identified on a functional cellular assay had more severe ECG conduction abnormalities and worse prognosis associated with earlier manifestations of lethal arrhythmic events (LAEs) (7.9%/year) than *in silico* algorithm-predicted SCN5A carriers (5.1%/year) or all BrS probands (2.5%/year). Importantly, non-LOF SCN5A variation carriers (*n* = 15) exhibited no LAEs during the follow-up period.^53^ Multivariate analysis demonstrated that only LOF SCN5A mutations and a history of aborted cardiac arrest were significant predictors of LAEs.^53^ Rare variations of non-SCN5A BrS-associated genes did not affect LAE-free survival curves. This study highlights that specific LOF SCN5A mutations could enable more refined risk stratification in BrS. Indeed, Ciconte *et al*.^54^ recently reported that SCN5A-positive BrS patients exhibited a larger epicardial area of fractionated, prolonged electrograms, and more frequent ECG late potentials. The presence of an SCN5A mutation explained >26% of the variation in epicardial abnormal substrate area. These data indicate a link between SCN5A determined epicardial conduction abnormalities and ventricular arrhythmias in BrS supporting the conduction reserve hypothesis but requires further refinement in determining the genetic architecture of pro-arrhythmic phenotypes in BrS.^55^

To conclude, The Year in Cardiovascular Medicine 2021—Arrhythmias has produced a diverse range of papers, with many highlighting key knowledge gaps for further investigation.

**Conflict of interest**: none declared.

## References

[1] Albert CM, Cook NR, Pester J, Moorthy MV, Ridge C, Danik JS, et al Effect of marine omega-3 fatty acid and vitamin D supplementation on incident atrial fibrillation: a randomized clinical trial. JAMA 2021;325:1061–1073. 33724323 10.1001/jama.2021.1489 PMC7967086

[2] Gencer B, Djousse L, Al-Ramady OT, Cook NR, Manson JE, Albert CM. Effect of long-term marine omega-3 fatty acids supplementation on the risk of atrial fibrillation in randomized controlled trials of cardiovascular outcomes: a systematic review and meta-analysis. Circulation 2021;144:1981–1990. 34612056 10.1161/CIRCULATIONAHA.121.055654 PMC9109217

[3] Csengeri D, Sprünker NA, Di Castelnuovo A, Niiranen T, Vishram-Nielsen JK, Costanzo S, et al Alcohol consumption, cardiac biomarkers, and risk of atrial fibrillation and adverse outcomes. Eur Heart J 2021;42:1170–1177. 33438022 10.1093/eurheartj/ehaa953 PMC7982286

[4] Wong JA, Conen D. Alcohol consumption, atrial fibrillation, and cardiovascular disease: finding the right balance. Eur Heart J 2021;42:1178–1179. 33438004 10.1093/eurheartj/ehaa955

[5] Lee SR, Choi EK, Jung JH, Han KD, Oh S, Lip GYH. Lower risk of stroke after alcohol abstinence in patients with incident atrial fibrillation: a nationwide population-based cohort study. Eur Heart J 2021;42:4759–4768. 34097040 10.1093/eurheartj/ehab315 PMC8651176

[6] Takahashi Y, Nitta J, Kobori A, Sakamoto Y, Nagata Y, Tanimoto K, et al Alcohol consumption reduction and clinical outcomes of catheter ablation for atrial fibrillation. Circ Arrhythm Electrophysiol 2021;14:e009770. 33999699 10.1161/CIRCEP.121.009770

[7] Tu SJ, Gallagher C, Elliott AD, Linz D, Pitman BM, Hendriks JML, et al Risk thresholds for total and beverage-specific alcohol consumption and incident atrial fibrillation. JACC Clin Electrophysiol 2021;7:1561–1569. 34330672 10.1016/j.jacep.2021.05.013

[8] Wang N, Sun Y, Zhang H, Wang B, Chen C, Wang Y, et al Long-term night shift work is associated with the risk of atrial fibrillation and coronary heart disease. Eur Heart J 2021;42:4180–4188. 34374755 10.1093/eurheartj/ehab505

[9] Wei D, Olofsson T, Chen H, Janszky I, Fang F, Ljung R, et al Death of a child and the risk of atrial fibrillation: a nationwide cohort study in Sweden. Eur Heart J 2021;42:1489–1495. 33515041 10.1093/eurheartj/ehaa1084 PMC8046501

[10] Smolderen KG, Burg MM. A broken heart after child loss. Eur Heart J 2021;42:1496–1498. 33585888 10.1093/eurheartj/ehab058

[11] Shahrbabaki SS, Linz D, Hartmann S, Redline S, Baumert M. Sleep arousal burden is associated with long-term all-cause and cardiovascular mortality in 8001 community-dwelling older men and women. Eur Heart J 2021;42:2088–2099. 33876221 10.1093/eurheartj/ehab151 PMC8197565

[12] Agerström J, Carlsson M, Bremer A, Herlitz J, Israelsson J, Årestedt K. Discriminatory cardiac arrest care? Patients with low socioeconomic status receive delayed cardiopulmonary resuscitation and are less likely to survive an in-hospital cardiac arrest. Eur Heart J 2021;42:861–869. 33345270 10.1093/eurheartj/ehaa954 PMC7897462

[13] Sondergaard KB, Wissenberg M, Gerds TA, Rajan S, Karlsson L, Kragholm K, et al Bystander cardiopulmonary resuscitation and long-term outcomes in out-of-hospital cardiac arrest according to location of arrest. Eur Heart J 2019;40:309–318. 30380021 10.1093/eurheartj/ehy687

[14] Jayawardana S, Mossialos E. The cost of prejudice for poorer people: understanding experiences of discrimination in cardiac arrest care. Eur Heart J 2021;42:870–872. 33374008 10.1093/eurheartj/ehaa1068

[15] Marijon E, Karam N, Jouven X. Cardiac arrest occurrence during successive waves of the COVID-19 pandemic: direct and indirect consequences. Eur Heart J 2021;42:1107–1109. 33543260 10.1093/eurheartj/ehab051 PMC7928959

[16] O’Shea CJ, Thomas G, Middeldorp ME, Harper C, Elliott AD, Ray N, et al Ventricular arrhythmia burden during the coronavirus disease 2019 (COVID-19) pandemic. Eur Heart J 2021;42:520–528. 33321517 10.1093/eurheartj/ehaa893 PMC7953962

[17] O’Shea CJ, Middeldorp ME, Thomas G, Harper C, Elliott AD, Ray N, et al Atrial fibrillation burden during the coronavirus disease 2019 pandemic. Europace 2021;23:1493–1501. 34077513 10.1093/europace/euab099 PMC8195127

[18] Stieglis R, Zijlstra JA, Riedijk F, Smeekes M, van der Worp WE, Tijssen JGP, et al Alert system-supported lay defibrillation and basic life-support for cardiac arrest at home. Eur Heart J 2021. 10.1093/eurheartj/ehab802 PMC9009403 34791171

[19] Schierbeck S, Hollenberg J, Nord A, Svensson L, Nordberg P, Ringh M, et al Automated external defibrillators delivered by drones to patients with suspected out-of-hospital cardiac arrest. Eur Heart J 2021. 10.1093/eurheartj/ehab498 34438449

[20] Glikson M, Nielsen JC, Kronborg MB, Michowitz Y, Auricchio A, Barbash IM, et al 2021 ESC Guidelines on cardiac pacing and cardiac resynchronization therapy. Eur Heart J 2021;42:3427–3520. 34586378 10.1093/eurheartj/ehab699

[21] Hindricks G, Potpara T, Dagres N, Arbelo E, Bax JJ, Blomstrom-Lundqvist C, et al 2020 ESC Guidelines for the diagnosis and management of atrial fibrillation developed in collaboration with the European Association for Cardio-Thoracic Surgery (EACTS): the Task Force for the diagnosis and management of atrial fibrillation of the European Society of Cardiology (ESC) Developed with the special contribution of the European Heart Rhythm Association (EHRA) of the ESC. Eur Heart J 2021;42:373–498. 32860505 10.1093/eurheartj/ehaa612

[22] Svendsen JH, Diederichsen SZ, Hojberg S, Krieger DW, Graff C, Kronborg C, et al Implantable loop recorder detection of atrial fibrillation to prevent stroke (the LOOP study): a randomised controlled trial. Lancet 2021;398:1507–1516. 34469766 10.1016/S0140-6736(21)01698-6

[23] Svennberg E, Friberg L, Frykman V, Al-Khalili F, Engdahl J, Rosenqvist M. Clinical outcomes in systematic screening for atrial fibrillation (STROKESTOP): a multicentre, parallel group, unmasked, randomised controlled trial. Lancet 2021;398:1498–1506. 34469764 10.1016/S0140-6736(21)01637-8

[24] Bonnesen MP, Frodi DM, Haugan KJ, Kronborg C, Graff C, Hojberg S, et al Day-to-day measurement of physical activity and risk of atrial fibrillation. Eur Heart J 2021;42:3979–3988. 34471928 10.1093/eurheartj/ehab597 PMC8497071

[25] Khurshid S, Weng LC, Al-Alusi MA, Halford JL, Haimovich JS, Benjamin EJ, et al Accelerometer-derived physical activity and risk of atrial fibrillation. Eur Heart J 2021;42:2472–2483. 34037209 10.1093/eurheartj/ehab250 PMC8291334

[26] Elliott AD, Middeldorp ME, Linz DK. The ins and outs of physical activity monitoring: implications for atrial fibrillation management. Eur Heart J 2021;42:3989–3991. 34468721 10.1093/eurheartj/ehab520

[27] Kotecha D, Bunting KV, Gill SK, Mehta S, Stanbury M, Jones JC, et al Effect of digoxin vs bisoprolol for heart rate control in atrial fibrillation on patient-reported quality of life: the RATE-AF randomized clinical trial. JAMA 2020;324:2497–2508. 33351042 10.1001/jama.2020.23138 PMC7756234

[28] Hallberg P, Lindbäck J, Lindahl B, Stenestrand U. Digoxin and mortality in atrial fibrillation: a prospective cohort study. Eur J Clin Pharmacol 2007;63:959–971. 17684738 10.1007/s00228-007-0346-9

[29] Turakhia MP, Santangeli P, Winkelmayer WC, Xu X, Ullal AJ, Than CT, et al Increased mortality associated with digoxin in contemporary patients with atrial fibrillation: findings from the TREAT-AF study. J Am Coll Cardiol 2014;64:660–668. 25125296 10.1016/j.jacc.2014.03.060 PMC4405246

[30] Whitbeck MG, Charnigo RJ, Khairy P, Ziada K, Bailey AL, Zegarra MM, et al Increased mortality among patients taking digoxin–analysis from the AFFIRM study. Eur Heart J 2013;34:1481–1488. 23186806 10.1093/eurheartj/ehs348

[31] Lim KT, Davis MJ, Powell A, Arnolda L, Moulden K, Bulsara M, et al Ablate and pace strategy for atrial fibrillation: long-term outcome of AIRCRAFT trial. Europace 2007;9:498–505. 17491103 10.1093/europace/eum091

[32] Wood MA, Brown-Mahoney C, Kay GN, Ellenbogen KA. Clinical outcomes after ablation and pacing therapy for atrial fibrillation: a meta-analysis. Circulation 2000;101:1138–1144. 10715260 10.1161/01.cir.101.10.1138

[33] Brignole M, Pentimalli F, Palmisano P, Landolina M, Quartieri F, Occhetta E, et al AV junction ablation and cardiac resynchronization for patients with permanent atrial fibrillation and narrow QRS: the APAF-CRT mortality trial. Eur Heart J 2021;42:4731–4739. 34453840 10.1093/eurheartj/ehab569

[34] Sutton R, Fedorowski A, Olshansky B, Gert van Dijk J, Abe H, Brignole M, et al Tilt testing remains a valuable asset. Eur Heart J 2021;42:1654–1660. 33624801 10.1093/eurheartj/ehab084 PMC8245144

[35] Brignole M, Russo V, Arabia F, Oliveira M, Pedrote A, Aerts A, et al Cardiac pacing in severe recurrent reflex syncope and tilt-induced asystole. Eur Heart J 2021;42:508–516. 33279955 10.1093/eurheartj/ehaa936 PMC7857694

[36] Simons SO, Elliott A, Sastry M, Hendriks JM, Arzt M, Rienstra M, et al Chronic obstructive pulmonary disease and atrial fibrillation: an interdisciplinary perspective. Eur Heart J 2021;42:532–540. 33206945 10.1093/eurheartj/ehaa822

[37] Romiti GF, Corica B, Pipitone E, Vitolo M, Raparelli V, Basili S, et al Prevalence, management and impact of chronic obstructive pulmonary disease in atrial fibrillation: a systematic review and meta-analysis of 4,200,000 patients. Eur Heart J 2021;42:3541–3554. 34333599 10.1093/eurheartj/ehab453

[38] Verdonschot JAJ, Merlo M, Dominguez F, Wang P, Henkens M, Adriaens ME, et al Phenotypic clustering of dilated cardiomyopathy patients highlights important pathophysiological differences. Eur Heart J 2021;42:162–174. 33156912 10.1093/eurheartj/ehaa841 PMC7813623

[39] Park S, Lee S, Kim Y, Lee Y, Kang MW, Kim K, et al Atrial fibrillation and kidney function: a bidirectional Mendelian randomization study. Eur Heart J 2021;42:2816–2823. 34023889 10.1093/eurheartj/ehab291

[40] Benn M . Atrial fibrillation and chronic kidney disease. Eur Heart J 2021;42:2824–2826. 34023873 10.1093/eurheartj/ehab301

[41] Whitlock RP, Belley-Cote EP, Paparella D, Healey JS, Brady K, Sharma M, et al Left atrial appendage occlusion during cardiac surgery to prevent stroke. N Engl J Med 2021;384:2081–2091. 33999547 10.1056/NEJMoa2101897

[42] Verma S, Bhatt DL, Tseng EE. Time to remove the left atrial appendage at surgery: LAAOS III in perspective. Circulation 2021;144:1088–1090. 34606300 10.1161/CIRCULATIONAHA.121.055825

[43] Toorop MMA, Chen Q, Tichelaar V, Cannegieter SC, Lijfering WM. Predictors, time course, and outcomes of persistence patterns in oral anticoagulation for non-valvular atrial fibrillation: a Dutch Nationwide Cohort Study. Eur Heart J 2021;42:4126–4137. 34269375 10.1093/eurheartj/ehab421 PMC8530535

[44] Oyama K, Giugliano RP, Berg DD, Ruff CT, Jarolim P, Tang M, et al Serial assessment of biomarkers and the risk of stroke or systemic embolism and bleeding in patients with atrial fibrillation in the ENGAGE AF-TIMI 48 trial. Eur Heart J 2021;42:1698–1706. 33760027 10.1093/eurheartj/ehab141 PMC8599897

[45] Krohn-Grimberghe M, Duerschmied D, Bode C. What do we learn by repeating the ABC? Eur Heart J 2021;42:1707–1709. 33769516 10.1093/eurheartj/ehab146

[46] Sulzgruber P, Doehner W, Niessner A. Personalized anti-thrombotic management of patients with non-valvular atrial fibrillation and a CHA2DS2-VASc score of 1—a statement of the ESC Working Group on Cardiovascular Pharmacotherapy and ESC Council on Stroke [corrected]. Eur Heart J 2021;42:541–543. 33496325 10.1093/eurheartj/ehaa1081

[47] Rosso R, Hochstadt A, Viskin D, Chorin E, Schwartz AL, Tovia-Brodie O, et al Polymorphic ventricular tachycardia, ischaemic ventricular fibrillation, and torsade de pointes: importance of the QT and the coupling interval in the differential diagnosis. Eur Heart J 2021;42:3965–3975. 33693589 10.1093/eurheartj/ehab138

[48] van der Werf C, Lambiase PD. Initiation and management of polymorphic ventricular tachycardia: history gone full circle. Eur Heart J 2021;42:3976–3978. 34378024 10.1093/eurheartj/ehab428

[49] Steinberg C, Davies B, Mellor G, Tadros R, Laksman ZW, Roberts JD, et al Short-coupled ventricular fibrillation represents a distinct phenotype among latent causes of unexplained cardiac arrest: a report from the CASPER registry. Eur Heart J 2021;42:2827–2838. 34010395 10.1093/eurheartj/ehab275

[50] Younis A, Goldberger JJ, Kutyifa V, Zareba W, Polonsky B, Klein H, et al Predicted benefit of an implantable cardioverter-defibrillator: the MADIT-ICD benefit score. Eur Heart J 2021;42:1676–1684. 33417692 10.1093/eurheartj/ehaa1057 PMC8088341

[51] Probst V, Goronflot T, Anys S, Tixier R, Briand J, Berthome P, et al Robustness and relevance of predictive score in sudden cardiac death for patients with Brugada syndrome. Eur Heart J 2021;42:1687–1695. 33289793 10.1093/eurheartj/ehaa763

[52] Delise P . Risk stratification in Brugada syndrome: the challenge of the grey zone. Eur Heart J 2021;42:1696–1697. 33428713 10.1093/eurheartj/ehaa1100

[53] Ishikawa T, Kimoto H, Mishima H, Yamagata K, Ogata S, Aizawa Y, et al Functionally validated *SCN5A* variants allow interpretation of pathogenicity and prediction of lethal events in Brugada syndrome. Eur Heart J 2021;42:2854–2863. 34219138 10.1093/eurheartj/ehab254

[54] Ciconte G, Monasky MM, Santinelli V, Micaglio E, Vicedomini G, Anastasia L, et al Brugada syndrome genetics is associated with phenotype severity. Eur Heart J 2021;42:1082–1090. 33221895 10.1093/eurheartj/ehaa942 PMC7955973

[55] Postema PG, Walsh R, Bezzina CR. Illuminating the path from genetics to clinical outcome in Brugada syndrome. Eur Heart J 2021;42:1091–1093. 33444429 10.1093/eurheartj/ehaa994 PMC7955964

[56] Nauffal V, Marstrand P, Han L, Parikh VN, Helms AS, Ingles J, et al Worldwide differences in primary prevention implantable cardioverter defibrillator utilization and outcomes in hypertrophic cardiomyopathy. Eur Heart J 2021;42:3932–3944. 34491319 10.1093/eurheartj/ehab598 PMC8497072

[57] Willems S, Borof K, Brandes A, Breithardt G, Camm AJ, Crijns H, et al Systematic, early rhythm control strategy for atrial fibrillation in patients with or without symptoms: the EAST-AFNET 4 trial. Eur Heart J 2022;43:1219–1230. 34447995 10.1093/eurheartj/ehab593 PMC8934687

